# Aspirin Eugenol Ester Ameliorates HFD-Induced NAFLD in Mice via the Modulation of Bile Acid Metabolism

**DOI:** 10.3390/ijms26157044

**Published:** 2025-07-22

**Authors:** Zhi-Jie Zhang, Qi Tao, Ji Feng, Qin-Fang Yu, Li-Ping Fan, Zi-Hao Wang, Wen-Bo Ge, Jian-Yong Li, Ya-Jun Yang

**Affiliations:** 1 College of Veterinary Medicine, Gansu Agricultural University, Lanzhou 730070, China; 18793107830@163.com; 2 Key Lab of New Animal Drug of Gansu Province, Key Lab of Veterinary Pharmaceutical Development of Ministry of Agriculture and Rural Affairs, Lanzhou Institute of Husbandry and Pharmaceutical Sciences of CAAS, Lanzhou 730050, China; taoqi19951224@163.com (Q.T.); fengji17520511105@163.com (J.F.); fanliping43t@163.com (L.-P.F.);

**Keywords:** aspirin eugenol ester (AEE), metabolomics, NAFLD, mice, high fat diet

## Abstract

Non-alcoholic fatty liver disease (NAFLD) is a highly prevalent condition worldwide and represents a major global health challenge. Pharmacological and pharmacodynamic results indicate that aspirin eugenol ester (AEE) performs various pharmacological activities. However, it is unclear whether AEE can ameliorate the NAFLD. This study investigated the ameliorative effects of AEE on glucose and lipid metabolism disorders by in vitro and in vivo experiments. In the cellular model, TC increased to 0.104 μmol/mg and TG increased to 0.152 μmol/mg in the model group, while TC decreased to 0.043 μmol/mg and TG decreased to 0.058 μmol/mg in the AEE group. In the model group, the area occupied by lipid droplets within the visual field was significantly elevated to 17.338%. However, the administration of AEE resulted in a substantial reduction in this area to 10.064%. AEE significantly reduced the lipid droplet area and TC and TG levels (*p* < 0.05), increased bile acids in the cells and in the medium supernatant (*p* < 0.05), and significantly up-regulated the expression of *LRH-1*, *PPARα*, *CYP7A1*, and *BSEP* mRNA levels (*p* < 0.05) compared to the model group. In the animal model, different doses of AEE administration significantly down-regulated the levels of TC, TG, LDL, GSP, and FBG (*p* < 0.05) compared to the high-fat-diet (HFD) group, and 216 mg/kg of AEE significantly improved hepatocellular steatosis, attenuated liver injury, and reduced the area of glycogen staining (*p* < 0.05). In the HFD group, the glycogen area within the visual field exhibited a significant increase to 18.250%. However, the administration of AEE resulted in a notable reduction in the glycogen area to 13.314%. Liver and serum metabolomics results show that AEE can reverse the metabolite changes caused by a HFD. The major metabolites were involved in seven pathways, including riboflavin metabolism, glycerophospholipid metabolism, tryptophan metabolism, primary bile acid biosynthesis, biosynthesis of unsaturated fatty acids, nicotinate and nicotinamide metabolism, and tryptophan metabolism. In conclusion, AEE had a positive regulatory effect on NAFLD.

## 1. Introduction

The process of lipid metabolism includes lipid synthesis, catabolism, digestion, absorption, and transport. Many factors affect lipid metabolism, including age, gender, HFD, genetics, and psychological stress [1]. With the gradual improvement in living standards, people’s eating habits have changed, and high-fat and high-sugar diets have gradually become more common. Long-term high-fat and high-sugar diets can lead to disturbances in glucose and lipid metabolism and significant increases in total cholesterol (TC), triglycerides (TGs), and fasting blood glucose levels [2]. Disorders of glucose and lipid metabolism represent significant risk factors for cardiovascular disease and are the hallmark of metabolic diseases, such as obesity, type 2 diabetes mellitus, hyperlipidemia, and NAFLD [3].

NAFLD is a chronic liver condition closely linked to metabolic dysfunction, featuring abnormal fat deposition in liver cells and is often accompanied by obesity, diabetes, and abnormal blood lipid levels and other metabolic disorders [4]. Statistical analysis indicated a global prevalence of NAFLD in the range of 6–35% [5]. Although the mortality rate of the disease was relatively low, it had been observed to gradually increase under the influence of factors such as genetics and changes in lifestyle. NAFLD is a significant threat to public health. Consequently, the development of safe and effective pharmaceuticals will be imperative to reduce NAFLD and could also ensure the health of animals.

Eugenol is the main component from the essential oils derived primarily from cinnamon, clove, and bay leaves, and it exhibits a wide range of pharmacological effects, including antioxidant, anti-cancer, and anti-inflammatory properties [6]. Aspirin, a non-steroidal anti-inflammatory drug, is utilized for the treatment and prevention of various diseases due to its analgesic, antipyretic, anti-inflammatory, antithrombotic, and antiviral properties [7]. However, the chemical structural instability of eugenol and the gastrointestinal side effects associated with aspirin significantly restrict their clinical applications. AEE is a novel compound synthesized by esterifying and modifying the carboxyl group of aspirin with the hydroxyl group of eugenol [8], which successfully increased the chemical–structural stability of the precursor drug and reduced the gastrointestinal side effects [9]. When AEE enters the body of animals, it is metabolized to salicylic acid and eugenol, which enhance the therapeutic effect by acting in a synergistic manner [10].

Metabolomics is a rapidly evolving technology that has been extensively applied in the study of diseases, including metabolic disorders, cancer, and others [11]. In a rat model of ulcerative colitis, the relationship among berberine, ulcerative colitis, and biological metabolites was elucidated through a metabolomic analysis of rat plasma [12]. In a rat model of primary liver cancer, differential metabolites were measured at various locations within the liver, thereby clarifying the role of walnut extract in antigenic liver cancer [13]. Utilizing metabolomics to investigate the composition of metabolites in the biological fluids of animal organs and target tissues allows for the identification of differential metabolites between healthy and pathological states. This approach effectively reflects changes in the metabolic profiles of animals and facilitates the detection of alterations in metabolic pathways. Such insights will enhance our understanding of the mechanisms by which drugs interact with disease models [14].

The primary aim of this study was to examine the protective effects of AEE on NAFLD and conduct a preliminary investigation into the molecular mechanisms through which AEE ameliorates this condition. The pharmacological effects of AEE on NAFLD were elucidated through both in vivo and in vitro experiments. Utilizing UPLC-QTOF-MS/MS metabolomics, this study preliminarily demonstrated that AEE may improve NAFLD by modulating bile acid metabolism. The regulation of bile acid metabolism by AEE was confirmed through the analysis of the expression levels of associated factors. This research aims to provide theoretical support and insights for the treatment of NAFLD with AEE.

## 2. Results

### 2.1. In Vitro Experiment

#### 2.1.1. Effects of AEE and High-Fat Inducers on AML-12 Cell Viability

**Figure 1:**
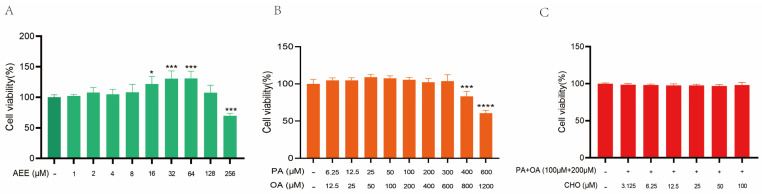
**Effects of AEE and high-fat inducers on AML-12 cell viability in mice.** (**A**) AEE: Aspirin eugenol ester, (**B**) PA: Palmitic acid + OA: Oleic acid, and (**C**) PA + OA + CHO: Cholesterol. Values are presented as the means ± SD where applicable (*n* = 5). * *p* < 0.05, *** *p* < 0.001, and **** *p* < 0.0001 compared to control group. One-way ANOVA followed by Duncan’s multiple comparisons.

Figure 1A shows that concentrations in the range of 1 μM to 128 μM AEE are non-toxic to AML-12 cells compared to the control. Specifically, 16 μM, 32 μM, and 64 μM AEE exhibit cell proliferation effects, whereas 256 μM AEE is toxic to AML-12 cells. Figure 1B shows that the concentrations of 6.25 μM palmitic acid (PA) + 12.5 μM oleic acid (OA) and 300 μM PA + 600 μM OA are non-toxic to AML-12 cells compared to the control, while concentrations of 400 μM PA + 800 μM OA and 600 μM PA + 1200 μM OA exhibit toxicity toward AML-12 cells. The results in Figure 1C show that 100 μΜ PA + 200 μΜ OA + 3.125 μΜ cholesterol (CHO) − 100 μΜ PA + 200 μΜ OA + 100 μΜ CHO are all non-toxic to AML-12 cells compared to the control. Consequently, AEE concentrations in this study were chosen as 16, 32, and 64 μM, while the concentrations of high-fat inducers were set at 100 μM PA, 200 μM OA, and 50 μM CHO.

#### 2.1.2. AEE Can Inhibit PA + OA + CHO-Induced Lipid Accumulation in AML-12 Cells

**Figure 2:**
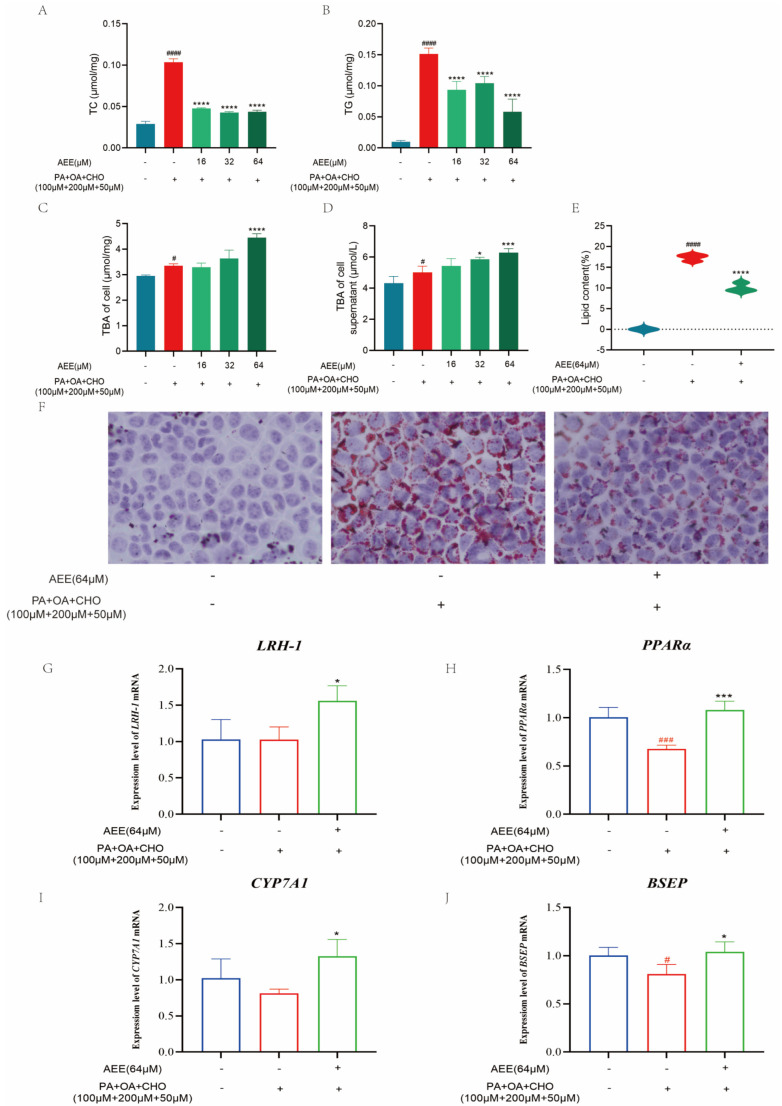
**AEE can inhibit PA + OA + CHO-induced lipid accumulation in AML-12 cells.** TC: Total cholesterol (**A**), TGs: Triglycerides (**B**), and TBA: Total bile acid (**C**) levels in AML12 cells from the indicated groups (*n* = 5). TBA (**D**) levels in AML12 cell supernatants of designated groups (*n* = 5). (**E**) Relative lipid content calculated using Image Pro plus 6.0 software (*n* = 3). (**F**) Representative images of oil red O (ORO) staining of different treated groups in AML-12 cells (400×) (*n* = 3). Expression of *LRH-1* (**G**)*, PPARα* (**H**), *CYP7A1* (**I**), and *BSEP* (**J**) mRNA levels in AML-12 cells (*n* = 3). Values are presented as the means ± SD where applicable. ^#^ *p* < 0.05, ^###^ *p* < 0.001, and ^####^ *p* < 0.0001 compared to control group. * *p* < 0.05, *** *p* < 0.001, and **** *p* < 0.0001 compared to model group. One-way ANOVA followed by Duncan’s multiple comparisons.

From Figure 2A,B, it can be seen that model group (100 μΜ PA + 200 μΜ OA + 50 μΜ CHO) significantly increases TC and TG levels compared to the control group, while 16, 32, and 64 μΜ AEE significantly reduced TC and TG levels compared to the model group. The model group significantly increased the intracellular total bile acid (TBA) content compared to the control group, while 64 μΜ of AEE could further increase the intracellular TBA content compared to the model group (Figure 2C). The model group could significantly increase the content of TBA in the supernatant of the cell culture medium compared to the control group, while 32 and 64 μΜ AEE further increased the content of TBA in the supernatant of the cell culture medium compared to the model group (Figure 2D). The results of oil red staining (Figure 2E,F) reveal that the area of lipid droplets in the visual field of the model group significantly increased to 17.338% compared to the control group, and 64 μΜ of AEE significantly reduced the area of lipid droplets in the visual field to 10.064% compared to the model group.

#### 2.1.3. AEE Promotes the mRNA Expression of Bile Acid Metabolism-Related Factors in AML-12 Cells

As shown in Figure 2G–J, the mRNA expression levels of *PPARα* and *BSEP* significantly decreased in the model group compared to the control group. In contrast, 64 μM AEE significantly up-regulated the expression of *LRH-1*, *PPARα*, *CYP7A1*, and *BSEP* compared to the model group.

### 2.2. In Vivo Experiment

#### 2.2.1. AEE Can Lower Blood Lipid Levels in HFD Mice

**Figure 3:**
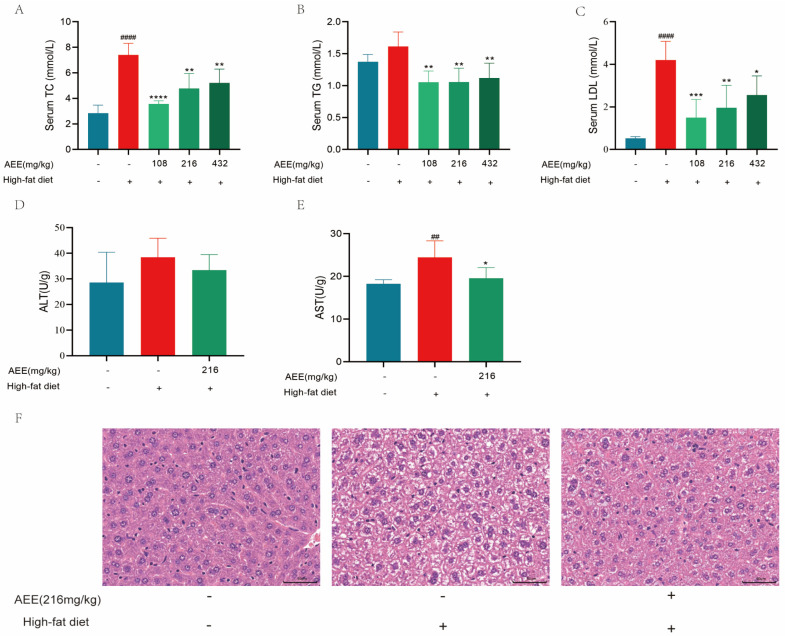
**AEE ameliorated high-fat diet-induced mouse dyslipidemia and liver injury**. Serum TC: serum total cholesterol (**A**), serum TGs: serum triglycerides (**B**), and serum LDL: serum low-density lipoprotein (**C**) levels from the indicated groups *(n* = 5). Hepatic ALT: Alanine aminotransferase (**D**) and AST: Aspartate aminotransferase (**E**) levels from the indicated groups (*n* = 5). (**F**) Representative images of H&E staining in liver sections from different groups (scale bars, 50 μm) (*n* = 3). Values are presented as the means ± SD where applicable. ^##^ *p* < 0.01, and ^####^ *p* < 0.0001 compared to control group. * *p* < 0.05, ** *p* < 0.01, *** *p* < 0.001, and **** *p* < 0.0001 compared to model group. One-way ANOVA followed by Duncan’s multiple comparisons.

From Figure 3A–C, it can be seen that a HFD significantly elevated the levels of TC, TG, and LDL in the blood of mice compared to the control group. In contrast, treatment with 108, 216, and 432 mg/kg of AEE significantly reduced the levels of TC, TG, and LDL in the blood of mice compared to the HFD group. The alanine aminotransferase (ALT) activity in the liver tissue of the mice was significantly increased in the HFD group compared to the control group, and the AEE at 216 mg/kg significantly reduced the ALT activity in the liver tissue of the mice compared to the HFD group, but it was not significant (Figure 3D). The HFD group significantly increased the aspartate aminotransferase (AST) activity in the liver tissues of mice compared to the control group, and AEE at 216 mg/kg significantly reduced the AST activity in the liver tissues of mice compared to the HFD group (Figure 3E). HE results (Figure 3F) show that in the control group, the peritoneum of the liver tissue and endothelium of the central vein are intact and the hepatocytes are arranged in a radial pattern. In the HFD group, a large number of hepatocytes were degenerated, and a large number of hepatocyte cytoplasm contained vacuoles of various sizes with nuclei floating in the center of the cells. In the AEE (216 mg/kg) group, a small number of hepatocytes with vacuolar degeneration were seen, and the structure of the hepatic sinusoids was normal with a clearer structure of the portal canalicular region.

#### 2.2.2. AEE Can Lower Blood Glucose Levels in HFD Mice

**Figure 4:**
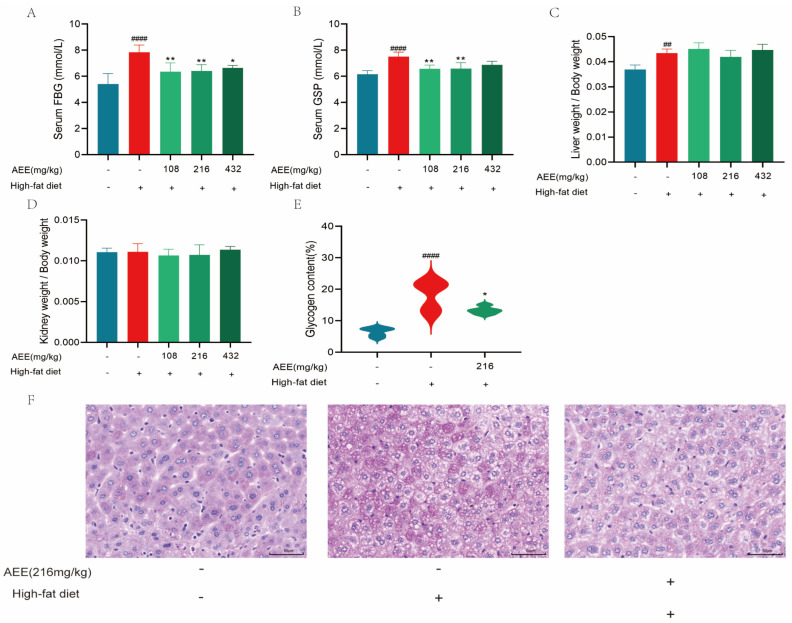
**AEE ameliorated glucose dysmetabolism in high-fat-fed mice.** FBG: Fasting blood glucose (**A**) and GSP: Glycosylated serum protein (**B**) levels from the indicated groups (*n* = 5). (**C**) Measurements of the ratio of liver weight to body weight (*n* = 5). (**D**) Measurements of the ratio of kidney weight to body weight (*n* = 5). (**E**) Relative glycogen content calculated using Image Pro plus 6.0 software (*n* = 3). (**F**) Representative images of PAS staining in liver sections from different groups (scale bars, 50 μm) (*n* = 3). Values are presented as the means ± SD where applicable. ^##^ *p* < 0.01, and ^####^ *p* < 0.0001 compared to control group. * *p* < 0.05, and ** *p* < 0.01 compared to model group. One-way ANOVA followed by Duncan’s multiple comparisons.

A HFD significantly increased the fasting blood glucose (FBG) levels in mice compared to the control group. Totals of 108, 216, and 432 mg/kg of AEE significantly decreased FBG levels in mice compared to the HFD group (Figure 4A). Glycosylated serum protein (GSP) content in mice was significantly increased in the HFD group compared to the control group, and 108 and 216 mg/kg of AEE significantly reduced the GSP content of mice compared to the HFD group (Figure 4B). The liver to body weight ratio of mice in the HFD group was significantly higher than that of the control group, and AEE did not reduce the liver to body weight ratio of mice compared to the HFD group (Figure 4C). The ratio of kidney to body weight showed no significant difference in each group (Figure 4D). The results of PAS staining show (Figure 4E,F) that the glycogen area in the visual field in the HFD group significantly increased to 18.250% compared to the control group, and AEE at 216 mg/kg significantly decreased the glycogen area in the visual field to 13.314% compared to the HFD group.

#### 2.2.3. There Was No Damage to the Stomach and Intestines of Mice in the AEE-M Group

**Figure 5:**
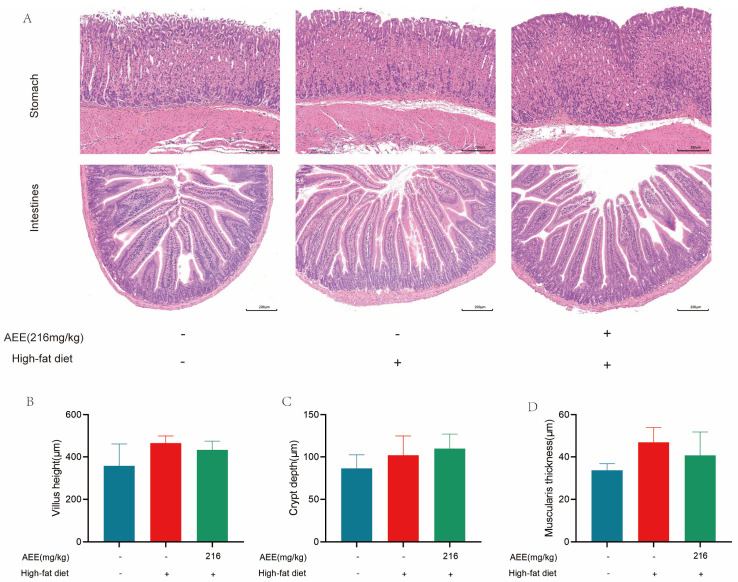
**Effects of AEE on the stomach and intestines of mice fed a high-fat diet.** (**A**) Representative images of H&E staining in stomach and intestines sections from different groups (scale bars, 200 μm) (*n* = 3). Measurements of the villus height (**B**), crypt depth (**C**), and muscularis thickness (**D**) (*n* = 3). Values are presented as the means ± SD where applicable. One-way ANOVA followed by Duncan’s multiple comparisons.

The results of Figure 5A show that in the control group, HFD group, and AEE-M group, the gastric tissue mucosal layer, submucosal layer, muscularis propria, and plasma membrane were structurally intact with no obvious hyperplasia, and the muscularis propria and ectodermal layer were normal with no obvious pathological changes. The mucosal and submucosal layers of the small intestine were structurally more complete with a regular cell arrangement, no obvious necrosis or sloughing, and no obvious inflammatory cell infiltration and hyperplasia in the outer membrane layer. Compared to the control group, the intestinal villus height, crypt depth, and muscularis thickness in HFD-group mice did not show significant differences (Figure 5B–D). Furthermore, when comparing AEE-M-group mice with the HFD group, there were also no significant differences observed in intestinal villi height, crypt depth, and muscle layer thickness.

### 2.3. Metabolomics Analysis Results

#### 2.3.1. Multivariate Statistical Analysis

**Figure 6:**
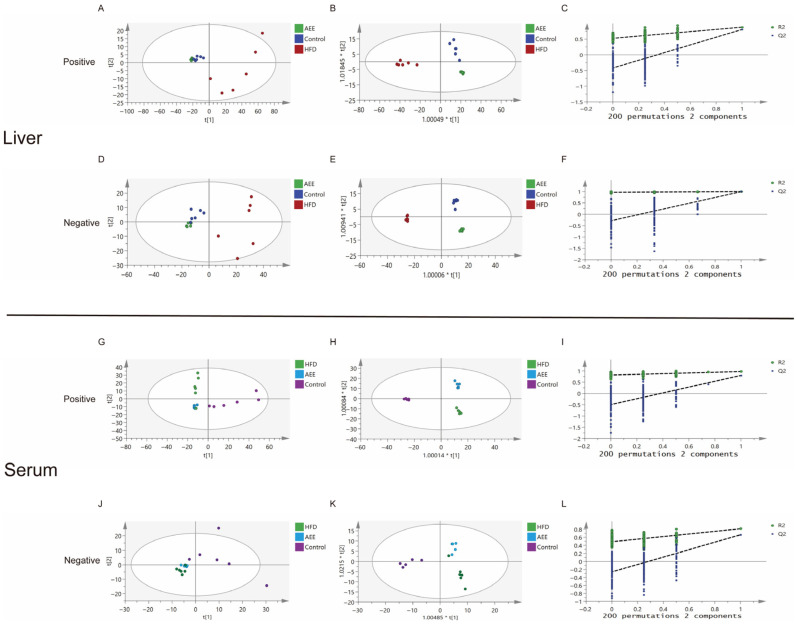
**Effect of AEE on the metabolomic profiles of the liver and serum of mice fed a high fat diet.** Plots of PCA scores of different groups in positive and negative modes. (**A**) Liver control group vs. model group vs. AEE group, ESI+: R^2^X = 0.845. (**D**) Liver control group vs. model group vs. AEE-M group, ESI−: R^2^X = 0.645. (**G**) Serum control group vs. model group vs. AEE group, ESI+: R^2^X = 0.844. (**J**) Serum control group vs. model group vs. AEE group, ESI−: R^2^X = 0.721. Plots of OPLS-DA scores of different groups in positive and negative modes. (**B**) Liver control group vs. model group vs. AEE group, ESI+: R^2^Y = 0.915, Q^2^ = 0.839. (**E**) Liver control group vs. model group vs. AEE group, ESI−: R^2^Y = 0.979, Q^2^ = 0.849. (**H**) Serum control group vs. model group vs. AEE group, ESI+: R^2^Y = 0.983, Q^2^ = 0.860. (**K**) Serum control group vs. model group vs. AEE group, ESI−: R^2^Y = 0.856, Q^2^ = 0.725. (**C**) Liver permutation test of OPLS-DA models in ESI+. (**F**) Liver permutation test of OPLS-DA models in ESI−. (**I**) Serum permutation test of OPLS-DA models in ESI+. (**L**) Serum permutation test of OPLS-DA models in ESI−.

Metabolite data from mice liver tissue and serum samples collected by UPLC-Q-TOF/MS were subjected to PCA and OPLS-DA. The metabolite data of the liver tissue samples from three groups were analyzed by PCA (Figure 6A,D) and the samples from three groups were well-separated, indicating that there were differences in the metabolomic profiles of the liver tissue samples from the control group, HFD group, and AEE group. The R^2^X values of the PCA model were 0.845 and 0.645 in the positive and negative ion modes, respectively. To further investigate the effect of AEE on the metabolic profiles of HFD mice, OPLS-DA was used to analyze the control, HFD, and AEE groups. The OPLS-DA model was validated using the replacement test. The parameter results of the OPLS-DA model for each group are shown in Figure 6B,C,E,F. The control, HFD, and AEE groups were compared with each other: ESI+: R^2^Y = 0.915, Q^2^ = 0.839 and ESI−: R^2^Y = 0.979, Q^2^ = 0.849. The results show a good model fit and predictive ability, and significant changes in the hepatic metabolic profiles of HFD mice after AEE administration.

The metabolite data of the serum samples from the three groups were analyzed by PCA (Figure 6G,J), and the samples from three groups were well-separated, suggesting that there were differences in the metabolomic profiles of the serum samples from the control, HFD, and AEE groups. The R^2^X values of the PCA model were 0.844 and 0.721 in the positive and negative ion modes, respectively. To further investigate the effect of AEE on the metabolic profiles of HFD mice, OPLS-DA was used to analyze the control, HFD, and AEE groups, and the OPLS-DA model was validated using the replacement test. The parameter results of the OPLS-DA model for each group are shown in Figure 6H,I,K,L. The control, HFD, and AEE groups were compared with each other: ESI+: R^2^Y = 0.983, Q^2^ = 0.860 and ESI−: R^2^Y = 0.856, Q^2^ = 0.725. The results show a good model fit and predictive ability, with significant changes in serum metabolic profiles in HFD mice after AEE administration.

#### 2.3.2. Analysis of Liver and Serum Metabolites in Mice

In the OPLS-DA model, differential metabolites were screened based on a VIP value greater than 1, a fold change (FC) less than 0.67 or greater than 1.50, and a *p*-value of less than 0.05. The identified differential metabolites were further analyzed using Target MS/MS scanning, and the secondary characteristic ion fragments of these metabolites were obtained for comparison with relevant databases.

Eight differential metabolites were finally examined in the analysis of liver samples, as shown in Table 1. Compared with the control group, a HFD could significantly reduce threonic acid, lysoPC (16:0/0:0), l-phosphoarginine, glycocholic acid, n-acetyl-l-methionine, l-tryptophan, and flavin mononucleotide. Compared to the control group, AEE could significantly increase threonic acid, lysoPC (16:0/0:0), l-phosphoarginine, glycocholic acid, taurocholic acid 3-sulfate, n-acetyl-l-methionine, l-tryptophan, and flavin mononucleotide. Compared with the HFD group, AEE was able to significantly increase threonic acid, lysoPC (16:0/0:0), l-phosphoarginine, taurocholic acid 3-sulfate, n-acetyl-l-methionine, l-tryptophan, and flavin mononucleotide.

Nine different metabolites were analyzed in the serum samples, as shown in Table 2. A HFD could significantly increase nudifloramide, eicosapentaenoic acid, n-stearoyl alanine, linoleyl carnitine, oleic acid, indoleacetaldehyde, lysoPC (20:2(11Z,14Z)), and 4-methylpentadecanoic acid levels compared with the control group. AEE was able to significantly increase nudifloramide, eicosapentaenoic acid, n-stearoyl alanine, linoleyl carnitine, oleic acid, indoleacetaldehyde, lysoPC (20:2(11Z,14Z)), and 4-methylpentadecanoic acid levels compared with the control group. AEE was able to significantly increase nudifloramide, eicosapentaenoic acid, n-stearoyl alanine, linoleyl carnitine, oleic acid, indoleacetaldehyde, lysoPC(20:2(11Z,14Z)), 4-methylpentadecanoic acid, and lysope (18:2(9Z,12Z)) compared with the HFD group.

#### 2.3.3. Analysis of Related Metabolic Pathways

**Figure 7:**
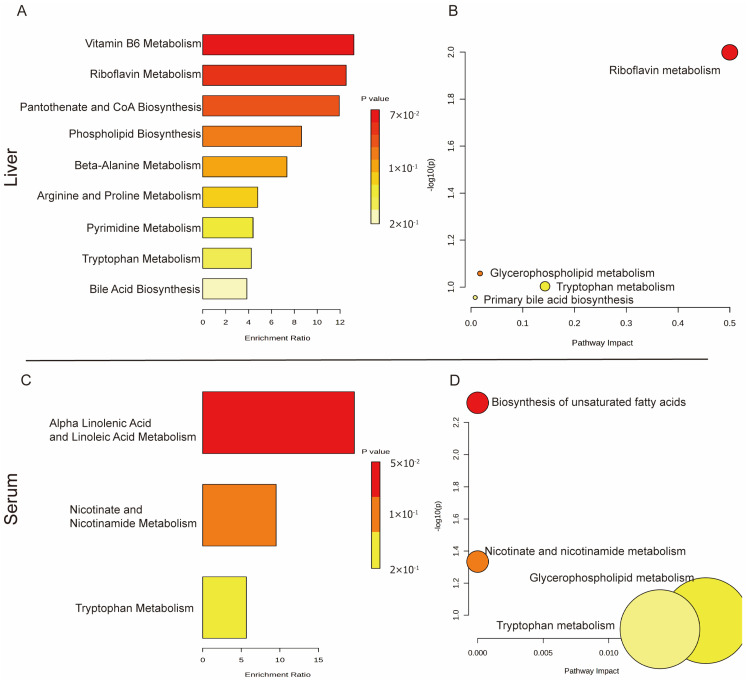
**Metabonomics analysis results.** (**A**) Liver metabolite enrichment analysis. (**B**) Liver metabolite pathway analysis. (**C**) Serum metabolite enrichment analysis. (**D**) Serum metabolite pathway analysis.

The differential metabolites screened in mice liver samples may be involved in the following pathways: riboflavin metabolism, glycerophospholipid metabolism, tryptophan metabolism, and primary bile acid biosynthesis (Figure 7A,B).

The differential metabolites screened in mice serum samples may be involved in the following pathways: biosynthesis of unsaturated fatty acids, nicotinate and nicotinamide metabolism, glycerophospholipid metabolism, and tryptophan metabolism (Figure 7C,D).

## 3. Discussion

AEE exhibits significant lipid-lowering effects, which can improve dyslipidemia in hyperlipidemic rats. AEE has been found to alleviate lipid accumulation and inflammation in macrophages induced by oxidized LDL [15,16]. Therefore, AEE shows good potential in the prevention of NAFLD. In this study, free fatty acids were utilized to induce AML-12 cells, thereby establishing a cell lipid deposition model. A mice model of NAFLD was induced using a HFD, which elucidated the pharmacological effects of AEE on NAFLD. Metabolomics was employed to investigate alterations in various metabolites and associated biological pathways in the liver and serum of mice, aiming to elucidate the potential mechanisms through which AEE mitigates NAFLD.

Long-term feeding of mice with a HFD leads to significant lipid metabolism disorders [17] and dyslipidemia [18], primarily characterized by increased TC, TG, and LDL levels compared to normal levels. Lotus seed protein improved hepatic lipid metabolism disorders in HFD mice, which significantly reversed the elevation of TC, TG, and LDL levels in HFD mice [19]. In this study, 6 weeks of high-fat feeding significantly increased the levels of TC, TG, and LDL in the blood of mice, whereas AEE administered to the groups significantly down-regulated the levels of TC, TG, and LDL, suggesting that AEE could ameliorate dyslipidemia in HFD mice.

The liver is an important organ involved in lipid metabolism. Animal organisms consume large amounts of lipids in their diet. However, when lipid levels exceed the liver’s ability to metabolize them, an abnormal accumulation of lipids in the liver will happen [20]. The excessive accumulation of lipids in the liver leads to hepatic steatosis, causing liver damage and the further development of liver fibrosis and hepatosclerosis [21]. High-fat feeding in mice leads to liver steatosis and liver damage [22], which is largely consistent with the results of this study. AST and ALT activity was significantly elevated in the liver of mice in the HFD group compared with the control group, which may be related to high-fat-feeding duration. The HE staining results demonstrate a substantial presence of fat vacuoles in the livers of HFD-group mice, indicating severe fat degeneration. In comparison, the AEE group exhibited a significant reduction in AST activity and a notable decrease in fat vacuoles within the liver tissues of the mice, which suggested that AEE could effectively ameliorate liver damage and liver steatosis induced by a prolonged HFD. However, the liver index did not show corresponding changes, which may be attributable to the short duration of the trial. This study also examined the pathological characteristics of the gastric and intestinal tissues, and no significant lesions were found in all test groups, and there was no significant difference in the height of the intestinal villi, the depth of the crypts, and the thickness of the muscularis propria, indicating that AEE at 216 mg/kg was non-invasive to the stomach and intestines of mice.

Blood glucose is a metabolic parameter that is an important indicator of the health of an organism, and GSP can respond to the average blood glucose level of the animal organism for 4–6 weeks, and examining changes in the GSP level is a key indicator of whether blood glucose has been effectively controlled [23,24]. A HFD can cause disturbances in glucose metabolism, which is consistent with this study [25,26,27]. Long-term high-fat diets affect glucolipid metabolism, which further impairs the function of the liver and other metabolic organs, alters insulin and β-cell function, and ultimately leads to the development of elevated blood glucose, hyperinsulinemia, and other glucose metabolism disorders [28]. These results show that fasting blood glucose and glycated serum protein levels are significantly higher in the HFD group of mice compared to the control group, and PAS staining showed a significant increase in the glycogen area, which could be significantly reversed in the AEE group. AEE significantly down-regulated fasting blood glucose and glycated serum protein levels in mice compared to the HFD group, and the glycogen area was also significantly reduced in the AEE group. Therefore, AEE could significantly improve glucose and lipid metabolism disorders induced by a HFD, indicating that AEE has the potential to treat NAFLD.

Under the in vitro conditions of this study, AEE was effective in reducing lipid accumulation in hepatocytes, thereby ameliorating lipid metabolism disorders. Reducing abnormal lipid accumulation in hepatocytes can be accomplished through mechanisms such as decreasing lipid synthesis and enhancing lipid transformation [29,30,31]. Cholesterol 7α-hydroxylase (CYP7A1) is the rate-limiting enzyme that converts cholesterol into bile acids. In the liver, bile acid production is the main metabolic pathway for cholesterol excretion. Alterations in bile acid metabolism induced by flammulina velutipes polysaccharides have beneficial effects on lipid metabolism in both obese mice and 3T3-L1 adipocytes [32], which suggests that regulating bile acid metabolism may be an effective strategy for improving NAFLD. This study assessed the bile acid content in AML-12 cells and the supernatant of the culture medium. Compared to the control group, the intracellular environment and culture medium supernatant in the model group exhibited a significant increase in bile acid content, which might be associated with excessive cholesterol uptake by the cells. Compared to the model group, the bile acid content in both the intracellular environment and medium supernatant in the AEE group showed varying degrees of increase. This suggested AEE could enhance bile acid metabolism and attenuate cholesterol accumulation in AML-12 cells under lipid deposition conditions.

Secondary bile acids exhibit a strong correlation with liver fat accumulation, given their ability to modulate lipid synthesis through the inhibition of Very Low-Density Lipoprotein (VLDL) secretion [33]. It has also been found that NAFLD can be effectively ameliorated by regulating gut flora and, thus, bile acid metabolism [34]. Liver receptor homolog-1 (LRH-1), a member of the NR5A nuclear receptor subfamily, is a potent transcriptional activator of CYP7A1. It binds to the CYP7A1 promoter as a monomer to regulate its expression [35]. Peroxisome proliferator-activated receptor α (PPARα), another member of the nuclear receptor family, is widely expressed in tissues, including the liver, heart, and kidney. The CYP7A1 promoter region contains a PPARα/retinoid X receptor (RXR) binding site. When PPARα forms a heterodimer with RXR, it binds to this regulatory element in the CYP7A1 promoter, thereby modulating CYP7A1 transcription [36]. Through this mechanism, PPARα affects the conversion of cholesterol to bile acids, thereby regulating lipid homeostasis. The bile salt export pump (BSEP) is located on the apical membrane and mediates the secretion of bile salts from hepatocytes into the bile; BSEP deficiency leads to hepatic cholestasis and liver injury [37]. To preliminarily investigate how AEE promotes bile acid metabolism, this study examined the expression of the above bile acid metabolism-related factors. AEE could significantly up-regulate the expression levels of *LRH-1*, *PPARα*, *CYP7A1*, and *BSEP* mRNA compared with the model group, suggesting that AEE may promote bile acid metabolism by regulating LRH-1 and PPARα, up-regulating the expression of CYP7A1, and transporting excess bile acids to the intestine and excreting them in the feces by up-regulating the expression of BSEP. These results suggest that AEE can promote bile acid metabolism by regulating the expression of LRH-1, PPARα, CYP7A1, and BSEP, thereby ameliorating NAFLD.

Using mice liver metabolomics in this study, we screened eight differential metabolites mainly involved in metabolic pathways, such as riboflavin metabolism, glycerophospholipid metabolism, tryptophan metabolism, and primary bile acid biosynthesis. In comparison to the control group, seven metabolites in the HFD group significantly decreased, while seven metabolites in the AEE group significantly increased compared to the HFD group. These findings indicate that AEE can effectively reverse the change in liver metabolites induced by a HFD.

The liver is a key organ and plays a crucial role in amino acid metabolism and maintaining amino acid homeostasis. A long-term HFD has been shown to cause a significant decrease in the amino acid levels in the liver of mice [38]. This finding aligns with the results of the present study and may be attributed to liver steatosis and injury induced by a HFD. In this study, a HFD induced amino acid metabolism disorders, resulting in a significant decrease in l-phosphoarginine, threonic acid, l-tryptophan, and n-acetyl-l-methionine. AEE could reverse this phenomenon, suggesting that AEE may improve amino acid metabolism disrupted by HFD-induced disorders. Methionine is an essential amino acid that plays a crucial role in regulating lipid metabolism, bile acid metabolism, and oxidative stress [39]. Tryptophan metabolism influences a diverse array of physiological functions, with its metabolites and derivatives positively contributing to the regulation of inflammation, immune responses, and oxidative stress [40]. Consequently, the elevation of methionine and tryptophan levels induced by AEE may mitigate inflammation and oxidative stress, thereby alleviating lipid metabolism disorders and liver injury associated with a HFD. Bile acids regulate glucose and lipid homeostasis by activating the expression of specific receptors. However, they are cytotoxic, and sulfation is crucial for their detoxification [41]. Taurocholic acid 3-sulfate, a sulfated bile acid, was significantly increased by AEE. This finding suggests that AEE not only reduces cholesterol accumulation in the liver by regulating bile acid metabolism, but also mitigates the cytotoxicity of bile acids through sulfation. Riboflavin, commonly referred to as vitamin B2, serves as a crucial cofactor for several key enzymes involved in lipid metabolism and the antioxidant system. It participates in various redox reactions in vivo as flavin mononucleotide and flavin adenine dinucleotide. A deficiency in riboflavin leads to significant lipid accumulation in the liver [42]. In the present study, flavin mononucleotide levels were significantly reduced in HFD mice, while they were significantly increased in mice from the AEE group. This suggested that AEE can ameliorate lipid metabolism disorders and oxidative imbalances induced by a HFD through the regulation of riboflavin metabolism.

Through mice serum metabolomics, this study identified a total of nine differential metabolites that were primarily involved in the pathways of biosynthesis of unsaturated fatty acids, nicotinate and nicotinamide metabolism, glycerophospholipid metabolism, and tryptophan metabolism. The results indicate that eight metabolites were significantly elevated in the HFD group compared with the control group. Serum levels of nudifloramide, eicosapentaenoic acid, n-stearoyl alanine, linoleyl carnitine, oleic acid, indoleacetaldehyde, lysoPC (20:2(11Z,14Z)), and 4-methylpentadecanoic acid were significantly elevated in mice after a HFD, which might be related to the compensatory response of the organism. AEE was able to significantly up-regulate the levels of nudifloramide, eicosapentaenoic acid, n-stearoyl alanine, linoleyl carnitine, oleic acid, indoleacetaldehyde, lysoPC (20:2(11Z,14Z)), and 4-methylpentadecanoic acid, lysope (18:2(9Z,12Z)) in the animals.

Oleic acid is a monounsaturated fatty acid and can improve lipid levels and blood glucose levels [43]. Oleic acid may improve inflammation by modulating arachidonic acid metabolism [44]. Eicosapentaenoic acid is a polyunsaturated fatty acid with anti-oxidant and anti-inflammatory properties [45]. In this study, AEE significantly increased the levels of oleic acid and eicosapentaenoic acid, suggesting that it may alleviate lipid metabolism disorders and inflammatory responses in HFD mice through the regulation of unsaturated fatty acid synthesis. Linoleyl carnitine, an acylcarnitine formed by the combination of fatty acids and carnitine, is a crucial fatty acid metabolite involved in energy metabolism and fatty acid oxidation. It facilitates the transport of fatty acids from the cytoplasm to the mitochondrial matrix for β-oxidation, thereby generating the energy necessary to sustain cellular activity [46]. In this study, AEE significantly increased the levels of linoleyl carnitine, indicating that AEE plays a role in the regulation of energy metabolism and fatty acid oxidation. Indoleacetaldehyde, a tryptophan metabolite, activates the aryl hydrocarbon receptor (AhR) and stimulates IL-22 production, thereby modulating intestinal homeostasis and maintaining gut microbiota balance [47]. A HFD disrupts gut microbiota homeostasis, and the restoration of this balance can ameliorate diet-induced lipid metabolic disorders [48]. In the present study, AEE significantly elevated indoleacetaldehyde levels, suggesting a positive effect of AEE on gut microflora homeostasis. LysoPE (18:2(9Z,12Z)), produced through the phospholipase A1-mediated hydrolysis of phosphatidylethanolamine involving fatty acid removal showed significantly decreased levels in both hepatic-injured and obese mice [49]. This reduction indicates that impaired lipid metabolism disrupts glycerophospholipid homeostasis. In this study, AEE significantly increased lysoPE (18:2(9Z,12Z)) levels in HFD mice, suggesting that AEE could ameliorate the disruption of glycerophospholipid metabolism caused by lipid metabolism disorders. Surprisingly, the HFD-fed mice in this study showed no significant reduction in LysoPE (18:2(9Z,12Z)) levels compared to the controls, potentially due to the duration of the HFD intervention. The metabolomic results suggest that AEE may act by regulating bile acid metabolism, amino acid metabolism, inflammatory response, oxidative stress, and other pathways. This study presents certain limitations. Specifically, only the expression of the relevant factors was validated. Future research will focus on validating the pertinent pathways using techniques such as agonists, inhibitors, knockdown, or overexpression to elucidate the mechanisms through which AEE ameliorates NAFLD.

## 4. Materials and Methods

### 4.1. Chemicals and Reagents

AML-12 cells were obtained from the American Type Culture Collection (ATCC, Manassas, VA, USA). AEE with a purity of 99.5% was synthesized at the Lanzhou Institute of Husbandry and Pharmaceutical Sciences of CAAS, Lanzhou, China. The complete medium for AML-12 cells (BNCC342626, BeNa Culture Collection, Suzhou, China) Was 0.05% Trypsin-EDTA (25300062, Gibco, Grand Island, NY, USA); phosphate-buffered saline (P1020, Solarbio, Beijing, China); Triton X-100 (T8200, Solarbio, Beijing, China); A BCA protein assay kit (PC0020, Solarbio, Beijing, China); Oil Red O Stain Kit (G1262, Solarbio, Beijing, China); cell counting kit-8 (C6005, NCM Biotech, Suzhou, China); Cellular Total Cholesterol Assay Kit (E1015, Applygen, Beijing, China); Triglyceride Content Assay Kit (E1013, Applygen, Beijing, China); Glycosylated Serum Protein Assay Kit (A037-2-1, Nanjing Jiancheng Bioengineering Institute, Nanjing, China); Total Cholesterol Assay Kit (A111-1-1, Nanjing Jiancheng Bioengineering Institute, Nanjing, China); Triglyceride Content Assay Kit (A110-1-1, Nanjing Jiancheng Bioengineering Institute, Nanjing, China); LDL Cholesterol Assay Kit (A113-1-1, Nanjing Jiancheng Bioengineering Institute, Nanjing, China); Total Bile Acid Kit (E003-2-1, Nanjing Jiancheng Bioengineering Institute, Nanjing, China); Aspartate Aminotransferase Activity Assay Kit (C010-2-1, Nanjing Jiancheng Bioengineering Institute, Nanjing, China); Alanine Aminotransferase Activity Assay Kit (C009-2-1, Nanjing Jiancheng Bioengineering Institute, Nanjing, China); TaKaRa MiniBEST Universal RNA Extraction Kit (9767, Takara, Nanjing, China); PrimeScript™ RT Master Mix (RR036A, Takara, Nanjing, China); TB Green^®^ Premix Ex Taq™ II FAST qPCR (CN830A, Takara, Nanjing, China); sodium carboxymethyl cellulose (30036328, Sinopharm Chemical Reagent Co. Ltd., Shanghai, China); cell culture plate (KG10006, Kejin, Shanghai, China); methanol (A456, Thermo Fisher Scientific Corporation, Waltham, MA, USA); and acetonitrile (A955, Thermo Fisher Scientific Corporation, Waltham, MA, USA). The HFD was a mixture of 77.8% standard diet, 10% egg yolk powder, 10% lard, 2% cholesterol, and 0.2% bile salts, with a lipid content of 41.5%, a carbohydrate content of 40.2%, and a protein content of 18.3%.

### 4.2. In Vitro Experiment

#### 4.2.1. Cell Culture

AML-12 cells were resuscitated and positioned in a cell culture incubator (5% CO_2_, 37 °C) with the complete medium for AML-12 cells (89% DMEM-H/F12 + 10% FBS + 1% ITS Liquid Media Supplement + 40 ng/mL Dexamethasone). Cells were observed under reversed biological microscope (Leica, Wetzlar, Germany), and when fusion reached approximately 90%, the cells were passaged with 0.05% Trypsin-EDTA for subsequent experiments.

#### 4.2.2. Cell Viability

The logarithmic growth phase of cells was assessed, and cells were inoculated onto 96-well plates. Different concentrations of AEE and high-fat inducer were added after waiting for the cells to completely adhere to the wall, and after incubation for 24 h, 10 μL of CCK8 reagent was added to each well. After incubation for 60 min in the cell culture incubator, OD values were detected at 450 nm to assess cell viability and select the appropriate doses of AEE and high-fat inducer. The data from this trial were analyzed using one-way analysis of variance (ANOVA). Values are presented as the means ± SD where applicable (*n* = 5).

#### 4.2.3. Treatment and Grouping of AML-12 Cells

AML-12 cells were inoculated onto 6-well plates at 1 × 10^5^ cells/mL and placed in a cell culture incubator (5% CO_2_, 37 °C) until the cells were completely attached to the wall. AML-12 cells were divided into 5 groups in the experimental, control, model, and AEE low, medium, and high administration groups. According to the cytotoxicity results, the AEE administration group was incubated with AML-12 cells full media containing different concentrations of AEE (16, 32, and 64 μM) for 24 h, and then with AML-12 cells full media containing high lipid inducers (100 μM PA + 200 μM OA + 50 μM CHO) for 24 h. The control group was incubated with AML-12 cells full media for 24 h and the culture medium was changed after 24 h. The model group was incubated with AML-12 cells full media for 24 h and then with AML-12 cells full media containing high lipid inducers (100 μM PA + 200 μM OA + 50 μM CHO) for 24 h.

#### 4.2.4. Detection of TC and TG Levels in Cells

After discarding the culture medium, harvest the cultured cells and wash them three times with PBS. Subsequently, lyse the cells using the kit-provided lysis solution, followed by centrifugation at 2000× *g* for 5 min at room temperature to collect the supernatant for testing. The TC and TG levels in AML-12 cells were determined in accordance with the TC and TG assay kit instructions. Protein concentrations were quantified for each sample and used to calibrate TC and TG measurements. The data from this trial were analyzed using one-way analysis of variance (ANOVA). Values are presented as the means ± SD where applicable (*n* = 5).

#### 4.2.5. TBA Content Test

The processed cells and cell supernatants were collected, and the TBA content in AML-12 cells and cell supernatants was determined using the TBA assay kit. The collected cell supernatants were directly assayed for TBA content. Then, we washed the cells three times with PBS, added 200 μL of 2% Triton X-100, and lysed them at 4 °C for 30 min. Collect the lysate to quantify intracellular TBA content. Protein concentrations were quantified for each sample and used to calibrate TBA measurements. The data from this trial were analyzed using one-way analysis of variance (ANOVA). Values are presented as the means ± SD where applicable (*n* = 5).

#### 4.2.6. Oil Red O Stain of Cells

After removing the cultured cells and discarding the medium, the cells were washed three times with PBS. Fixative was then added and incubated for 25 min at room temperature. Following discarding the fixative, the cells were washed twice with distilled water and treated with 60% isopropanol for 25 s. After discarding the isopropanol, an oil red staining solution was added to immerse the cells for 20 min. The staining solution was then discarded, and the cells were treated with 60% isopropanol for 25 s, followed by five washes with distilled water. Subsequently, a hematoxylin staining solution was added to stain nuclei for 2 min. After discarding the staining solution, the cells were washed five times with distilled water, incubated with buffer for 1 min, and the buffer was discarded. Finally, distilled water was added and the lipid droplet size was observed under a microscope.

The Image-Pro Plus 6.0 image analysis system was utilized to quantify the area of lipid droplet expression in all collected images. The percentage of the lipid droplet expression area was calculated by dividing the area of lipid droplet expression by the total field of view area (measured in pixels). The data from this trial were analyzed using one-way analysis of variance (ANOVA). Values are presented as the means ± SD where applicable (*n* = 3).

#### 4.2.7. RT-qPCR

Total RNA from AML-12 cells was extracted according to the kit instructions, and the extracted total RNA was reverse transcribed to cDNA, followed by quantitative PCR using the SYBR Green reagent. The PCR conditions were as follows: 95 °C for 5 s followed by 60 °C for 10 s (40 cycles). The samples were quantified using the comparative Ct method for the relative quantification of gene expression, normalized to *β-actin*. PCR primer sequences are listed in Table 3. The data from this trial were analyzed using one-way analysis of variance (ANOVA). Values are presented as the means ± SD where applicable (*n* = 3).

### 4.3. In Vivo Experiment

#### 4.3.1. Animal Experiment and Study Design

Thirty male pathogen-free C57BL/6J mice (8 weeks old, 28–30 g) from the Lanzhou Veterinary Research Institute, Chinese Academy of Agricultural Sciences (Lanzhou, China), were used. The mice were housed in an SPF-rated laboratory with controlled relative humidity (55–65%), a 12 h light/dark cycle, and a temperature of 24 ± 2 °C. Mice had free access to food and water. All experimental protocols and procedures were approved by the Institutional Animal Care and Use Committee of the Lanzhou Institute of Husbandry and Pharmaceutical Science, Chinese Academy of Agricultural Sciences (Approval Date: 2024-50). Animal welfare and experimental procedures were conducted in strict accordance with the US National Institutes of Health Guidelines for the Care and Use of Laboratory Animals.

**Figure 8:**
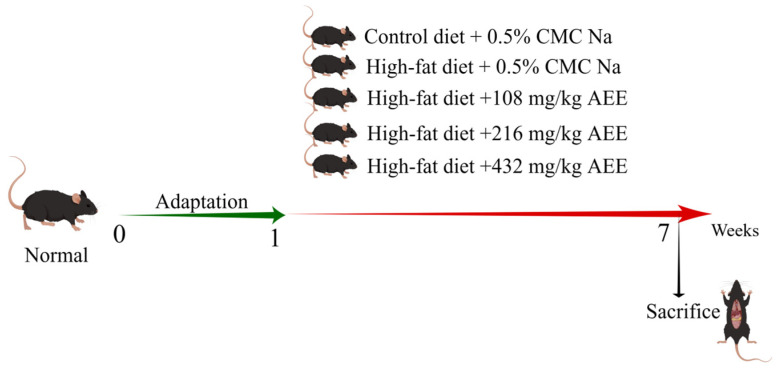
Grouping and handling of mice in this experiment.

Thirty pathogen-free male C57BL/6J mice were randomly allocated to five experimental groups, each consisting of six mice: the control group (control), the model group (HFD), the AEE low-dose group (AEE-L), the AEE medium-dose group (AEE-M), and the AEE high-dose group (AEE-H), as shown in Figure 8. The mice were housed in M-3-type cages, measuring 318 × 202 × 135 mm, with six mice per cage to ensure sufficient space for movement. Following a one-week acclimatization period, the control group was fed a normal diet, while the HFD group received a HFD. The AEE-L, AEE-M, and AEE-H groups were concurrently administered a HFD and varying doses of AEE: 108 mg/kg, 216 mg/kg, and 432 mg/kg, respectively. AEE suspensions were prepared using 0.5% CMC-Na, and mice in the AEE groups received AEE via gavage once daily for six weeks. To control for the effects of the vehicle (CMC-Na), mice in the control and HFD groups were administered an equivalent volume of CMC-Na as those in the AEE groups. Previous research has determined that the optimal dose of AEE for rats is 54 mg/kg [15], which, when adjusted using the body surface area (BSA) method, corresponds to 108 mg/kg for mice. Similarly, the optimal dose of aspirin for HFD mice is 120 mg/kg [50], which, when converted by molar equivalence, equates to 217 mg/kg for mice. Consequently, the current study employed doses of 108 mg/kg (low), 216 mg/kg (medium), and 432 mg/kg (high) for AEE, with a dose ratio of 1:2:4.

After the last gavage, mice were fasted for 12 h. Mice were anesthetized by intraperitoneal injection of 60 mg/kg sodium pentobarbital, blood was collected from the orbits, and serum was isolated for further analysis. Mouse liver tissues were carefully collected, snap-frozen in liquid nitrogen, and stored at −80 °C.

#### 4.3.2. Measurement of Fasting Blood Glucose and Glycosylated Serum Protein Levels

After the last gavage, mice were fasted for 12 h. Blood was collected from the tail and fasting blood glucose levels were measured using a Roche ACCU-CHEK ^®^ Instant blood glucose meter (Roche, Basel, Switzerland). Thaw the serum at room temperature, take 10 μL of serum and mix it with 200 μL of NBT color developer, and place in a water bath at 37 °C for 15 min. Then, 10 μL of stabilizer was added, and after shaking well, the absorbance was detected at 530 nm to calculate the glycosylated serum protein content. The data from this trial were analyzed using one-way analysis of variance (ANOVA). Values are presented as the means ± SD where applicable (*n* = 5).

#### 4.3.3. Measurement of the Organ Index

Body weight was recorded prior to execution. After execution, the spleen and liver were removed and weighed. The organ index was calculated using the following formula: Organ index = tissue weight (g)/body weight (g). The data from this trial were analyzed using one-way analysis of variance (ANOVA). Values are presented as the means ± SD where applicable (*n* = 5).

#### 4.3.4. Blood Lipid Test

Allow the serum to thaw at ambient room temperature. Subsequently, extract 2.5 μL of the serum and combine it with 250 μL of the working solution. Incubate the mixture at 37 °C for a duration of 10 min. The absorbance was measured at a wavelength of 500 nm, and the concentrations of TC and TG were subsequently calculated. Combine 2.5 μL of serum with 180 μL of reagent I, incubate at 37 °C for 5 min, and record the absorbance at 600 nm as A1. Next, add 60 μL of reagent II, incubate again at 37 °C for 5 min, and measure the absorbance at 600 nm as A2. Determine the difference of A2-A1 and calculate the LDL amount as per the instructions. The data from this trial were analyzed using one-way analysis of variance (ANOVA). Values are presented as the means ± SD where applicable (*n* = 5).

#### 4.3.5. Liver Function Test

Allow the serum to thaw at ambient room temperature. Combine 5 μL of serum with 20 μL of reagent I; incubate at 37 °C for 30 min. An additional 20 μL of reagent II was introduced, and the reaction proceeded at 37 °C for 20 min. Subsequently, 200 μL of sodium hydroxide was added, and the mixture was incubated at room temperature for 15 min. The absorbance was then measured at 505 nm. The activities of ALT and AST were calculated in accordance with the provided instructions. The data from this trial were analyzed using one-way analysis of variance (ANOVA). Values are presented as the means ± SD where applicable (*n* = 5).

#### 4.3.6. Histopathological Analysis

Liver, duodenum, and stomach tissues were collected after the mice were sacrificed, fixed with 4% paraformaldehyde, dehydrated in a fully automated dehydrator, embedded, and sectioned. By combining the results of the above tests, the control group, HFD group, and AEE-M group were selected for HE staining and PAS staining, and the tissue lesions and glycogen distribution were observed by light microscopy. Three mice were randomly selected for each experimental group. For each mouse, a single section was prepared, and three images were obtained from each section.

The HE staining process involved 5 min of hematoxylin staining, followed by washing with tap water until colorless. Differentiation was performed with alcoholic hydrochloric acid for about 3 s, then rinsed with tap water. A weakly alkaline solution was used to return the color to blue, followed by another tap water wash. Eosin was applied for 3 min, then the sample underwent gradient alcohol dehydration, clearing, and sealing with neutral gum.

The PAS staining procedure involved several steps. Initially, the sample was treated with a PAS oxidizer for 5 min. Subsequently, it was immersed in Schiff reagent for 10 min. After the Schiff reagent was removed, the sample was rinsed with running water for 10 min. Next, the sample was stained with hematoxylin staining solution for 2 min to stain the nuclei. Following this, hydrochloric acid differentiation was performed, and the sample was washed with water. Once the sample returned to a blue hue, it was washed with water for an additional 3 min. The process concluded with gradient alcohol dehydration, clearing, and sealing with neutral gum.

Additionally, glycogen was quantified based on the percentage of the positive expression area. The Image-Pro Plus 6.0 image analysis system was employed to determine the area of positive expression (Area) within the captured images. The percentage of the positive expression area was calculated as the ratio of the area of positive expression to the field of view area (measured in pixels). This work was carried out by Chengdu Lilai Biotechnology Co, Ltd. (Chengdu, China). The data from this trial were analyzed using one-way analysis of variance (ANOVA). Values are presented as the means ± SD where applicable (*n* = 3).

### 4.4. Metabolomics Analysis

#### 4.4.1. Sample Processing

Based on the experimental results, the control, HFD, and AEE-M groups were subjected to metabolomics analysis.

Serum samples were thawed at room temperature [51]. For protein precipitation, 200 μL of serum was mixed with acetonitrile at a volume ratio of 3:1. After vortex-mixing vigorously for 1 min, the mixture was allowed to stand at room temperature for 10 min. Following the centrifugation of the supernatant at 14,000 rpm for 10 min at 4 °C, the resulting supernatant was filtered through a 0.22 μm membrane filter to prepare the test sample. From each prepared sample, 30 μL aliquots were separately aspirated and combined to create a quality control (QC) sample.

Liver tissue samples (200 mg) were homogenized in 2 mL of methanol/water (4:1, *v*/*v*) using a tissue homogenizer, followed by vortex-mixing for 1 min and ultrasonic extraction for 8 min. The mixture was then incubated on ice for 10 min and centrifuged at 15,000 rpm (4 °C) for 10 min. A 1.6 mL aliquot of the supernatant was collected, evaporated to dryness, and reconstituted in 200 μL of methanol/water (4:1, *v*/*v*) as the test sample [52]. For quality control (QC), 30 μL was separately aspirated from each prepared sample and pooled.

#### 4.4.2. UPLC-QTOF-MS/MS Conditions

Chromatographic separation was performed on an Agilent 1290 UPLC system (Agilent Technologies, Santa Clara, CA, USA) using an ACQUITY UPLC HSS T3 column (2.1 × 150 mm, 1.8 μm) maintained at 35 °C. The mobile phase consisted of 0.1% (*v*/*v*) aqueous formic acid (A) and 0.1% (*v*/*v*) formic acid in acetonitrile (B), delivered at a flow rate of 0.3 mL/min. Sample injection volume was 2 μL, with the autosampler temperature set to 4 °C.

For serum samples, the gradient elution of A was as follows: 95% A from 0 to 3 min, 95–35% A from 3 to 4 min, 35–30% A from 4 to 7 min, 30–15% A from 7 to 10 min, 15–10% A from 10 to 15 min, 10–5% A from 15 to 18 min, and 18 to 20 min kept at 95% A.

For liver samples, the gradient elution of A was as follows: 98% A from 0 to 2 min, 98–55% A from 2 to 11 min, 55–30% A from 11 to 15 min, 30–2% A from 15 to 22 min, and 2% A from 22 to 27 min.

High-resolution mass spectrometry analysis was performed using an Agilent 6530 Q-TOF system (Agilent Technologies, Santa Clara, CA, USA) with electrospray ionization (ESI) in the positive or negative mode. The optimized parameters were set as follows: mass range of *m*/*z* 50–1000 (centroid mode), scan rate of 1 spectrum/s, desolvation gas flow rate of 10 L/min at 350 °C, nebulizer pressure of 45 psig, fragment voltage of 135 V, skimmer voltage of 65 V, and capillary voltages of 4.0 KV (positive mode) and 3.5 KV (negative mode).

#### 4.4.3. Metabolomics Data Analysis

Untargeted metabolomic profiling was performed using a UPLC/Q-TOF-MS system (Agilent Technologies) for raw data acquisition. MS-DIAL software (v4.38) was employed for data preprocessing, including noise filtering, baseline correction, peak identification, data reduction, and normalization, resulting in a high-quality feature table comprising *m*/*z* values, retention times, and peak areas. Multivariate statistical analyses were conducted in Simmca 14.1, with the results visualized via principal component analysis (PCA) and orthogonal partial least squares discriminant analysis (OPLS-DA) score plots. Differential metabolites were screened based on the following criteria: variable importance in projection (VIP) >1, fold change (FC) thresholds of <0.67 or >1.50, and statistical significance (*p* < 0.05). Subsequently, targeted MS/MS analysis (UPLC/Q-TOF-MS) was applied to validate screened metabolites, acquiring precise fragment ion spectra aligned to their *m*/*z* and retention times. Metabolite identification was achieved through database matching against HMDB (http://www.hmdb.ca/, accessed 10 May 2025), METLIN (https://metlin.scripps.edu, accessed 10 May 2025), and KEGG (https://www.genome.jp/kegg/, accessed 10 May 2025). Finally, pathway enrichment analysis was performed using MetaboAnalyst 5.0 (http://www.metaboanalyst.ca/, accessed 10 May 2025) by importing the differential metabolite list.

### 4.5. Statistical Analysis

All data are presented as means ± SD. Data were analyzed using GraphpadPrism 9.0 and one-way ANOVA was used to test the significance of differences between groups, with *p* < 0.05 indicating statistical significance. ^#^ *p* < 0.05, ^##^ *p* < 0.01, ^###^ *p* < 0.001, and ^####^ *p* < 0.0001 compared to the control group. * *p* < 0.05, ** *p* < 0.01, *** *p* < 0.001, and **** *p* < 0.0001 compared to the model group.

## 5. Conclusions

AEE can prevent the occurrence and progression of non-alcoholic fatty liver disease by improving dyslipidemia, reducing lipid deposition in the liver, and regulating bile acid metabolism. Additionally, AEE may mitigate the damage caused by disorders in NAFLD by regulating inflammation, oxidative stress, and amino acid metabolism.

## Figures and Tables

**Table 1:** Liver tissue homogenates as potential biomarkers.

RT	VIP	Formula	Metabolites	SM	ME (ppm)	*m*/*z*	Fold Change		
							AEE/C	HFD/C	AEE/HFD
3.177	1.15	C_4_H_8_O_5_	Threonic acid	ESI+	3	137.0448	5.477 **	0.297 ***	18.424 ***
18.102	1.13	C_24_H_50_NO_7_P	LysoPC (16:0/0:0)	ESI+	15	496.3325	3.355 **	0.232 ***	14.441 ***
9.116	1.10	C_6_H_15_N_4_O_5_P	L-Phosphoarginine	ESI+	8	255.0832	4.189 **	0.173 ***	24.159 ***
11.678	1.28	C_26_H_43_NO_6_	Glycocholic acid	ESI−	3	464.3031	0.003 ***	0.002 **	—
10.444	1.10	C_26_H_45_NO_10_S_2_	Taurocholic acid 3-sulfate	ESI−	5	296.6185	10.872 **	—	18.548 **
7.483	1.23	C_7_H_13_NO_3_S	N-Acetyl-L-methionine	ESI−	3	190.0550	3.405 **	0.552 *	6.170 ***
7.026	1.28	C_11_H_12_N_2_O_2_	L-Tryptophan	ESI−	2	407.1731	1.650 *	0.445 ***	3.708 ***
7.543	1.15	C_17_H_21_N_4_O_9_P	Flavin mononucleotide	ESI−	4	455.0991	5.098 *	0.167 **	30.445 **

RT, retention time; ME, mass error in ppm; +, metabolites identified in positive mode; −, metabolites identified in negative mode. Metabolites identified in both positive and negative modes; * *p* < 0.05, ** *p* < 0.01, and *** *p* < 0.001. AEE/C: AEE vs. Control; HFD/C: HFD vs. Control; AEE/HFD: AEE vs. HFD.

**Table 2:** Serum tissue homogenates as potential biomarkers.

RT	VIP	Formula	Metabolites	SM	ME (ppm)	*m*/*z*	Fold Change		
							AEE/C	HFD/C	AEE/HFD
2.999	1.19	C_7_H_8_N_2_O_2_	Nudifloramide	ESI+	6	153.0649	47.155 **	15.418 *	3.058 *
11.784	1.36	C_20_H_30_O_2_	Eicosapentaenoic acid	ESI+	4	303.2305	13.094 ***	3.201 ***	4.091 *
15.443	1.20	C_21_H_41_NO_3_	N-Stearoyl Alanine	ESI+	5	356.3142	71.990 **	13.619 *	5.286 **
10.284	1.27	C_25_H_46_NO_4_	Linoleyl carnitine	ESI+	3	424.3408	54.090 **	10.806 **	5.005 *
17.655	1.21	C_18_H_34_O_2_	Oleic acid	ESI+	3	283.2624	44.790 **	7.946 **	5.637 **
3.129	1.42	C_10_H_9_NO	Indoleacetaldehyde	ESI+	6	160.0748	41.461 ***	8.631 ***	4.804 *
11.882	1.24	C_28_H_54_NO_7_P	LysoPC(20:2(11Z,14Z))	ESI+	6	548.3677	15.655 **	2.485 *	6.301 **
17.274	1.35	C_16_H_32_O_2_	4-methylpentadecanoic acid	ESI+	7	257.2457	29.558 ***	4.214 ***	7.014 **
10.469	1.22	C_23_H_44_NO_7_P	LysoPE(18:2(9Z,12Z))	ESI−	6	476.2753	—	—	2.005 *

RT, retention time; ME, mass error in ppm; +, metabolites identified in positive mode; −, metabolites identified in negative mode. Metabolites identified in both positive and negative modes; * *p* < 0.05, ** *p* < 0.01, and *** *p* < 0.001. AEE/C: AEE vs. Control; HFD/C: HFD vs. Control; AEE/HFD: AEE vs. HFD.

**Table 3:** Primer sequences used in the experiments.

Gene	Forward Primer	Reverse Primer
*Mouse LRH-1*	ATGTCTCAAGTTCCTGGTGCTGTTC	AGTCTTCTGCCTGCTTGCTGATTG
*Mouse* PPA*Rα*	CCAAGGCTATCCCAGGCTTTGC	GATGTCACAGAACGGCTTCCTCAG
*Mouse CYP7A1*	AGGTCTCTGAACTGATCCGTCTACG	GCGTCTTAGCCTTCTCCATGTCATC
*Mouse BSEP*	GAAGCCATTGCCGACCAGATG	GACTGACAGCGAGAATCACCAAG
*Mouse β-actin*	TACTGCTCTGGCTCCTAGCA	CGGACTCATCGTACTCCTGC

## Data Availability

The data that support the findings of this study are available from the corresponding authors upon reasonable request. Some data may not be made available because of privacy or ethical restrictions.
